# Supplementary figures and images for: Innovation in Nucleotide-Binding Oligomerization-Like Receptor and Toll-Like Receptor Sensing Drives the Major Histocompatibility Complex-II Free Atlantic Cod Immune System
Source: Front Immunol. 2020 Dec 11;11:609456. doi: 10.3389/fimmu.2020.609456 (PMC7759675; doi:10.3389/fimmu.2020.609456)

*S. cerevisiae*

#1

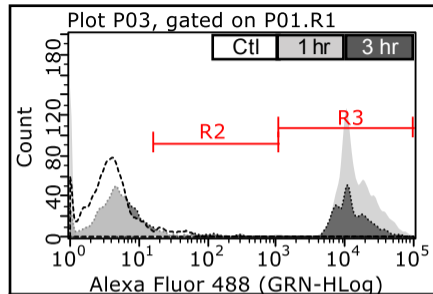

#2

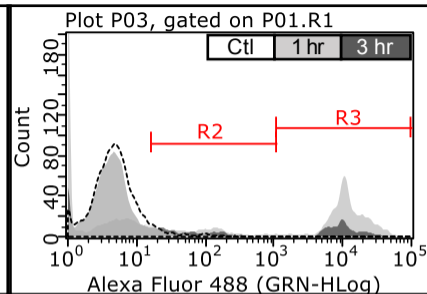

#3

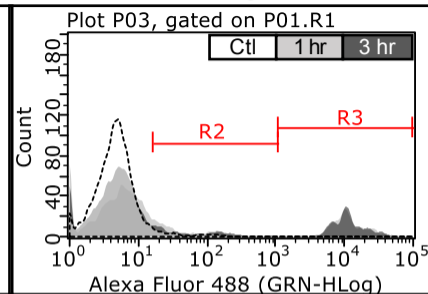

#4

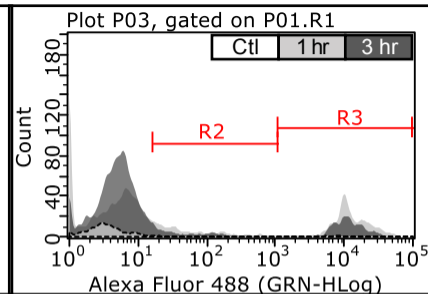

*E. coli*

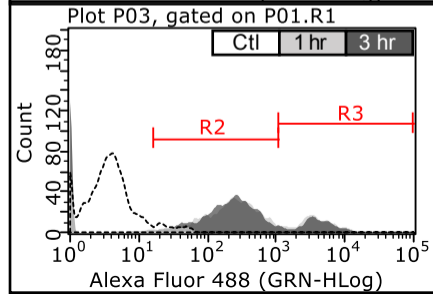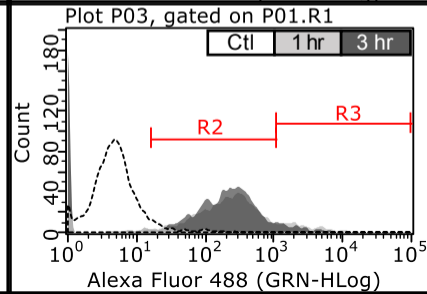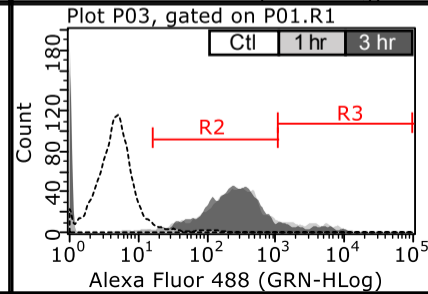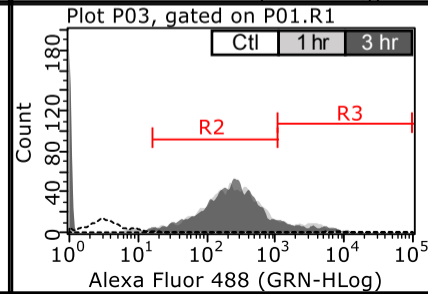

Supplement: Supplementary Data Sheet 1 — Differential expression analysis and GOs/KEGGs enrichment output. [file DataSheet_1.pdf]

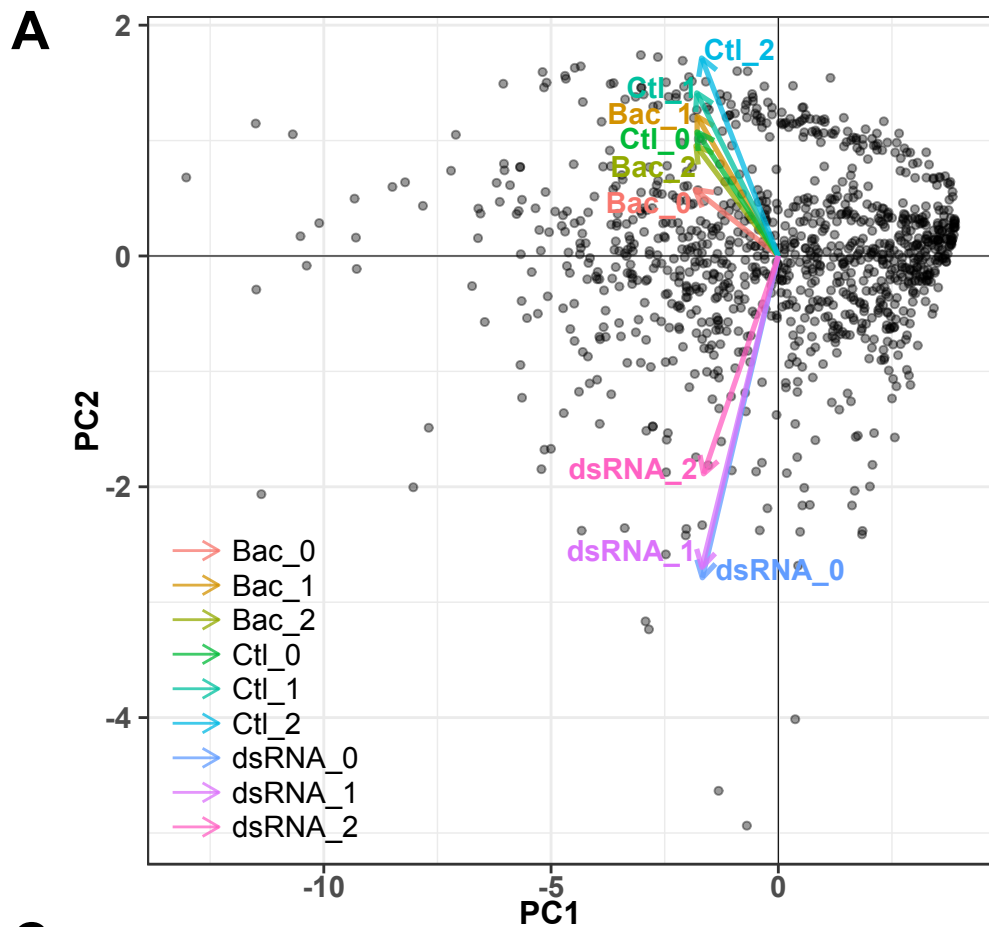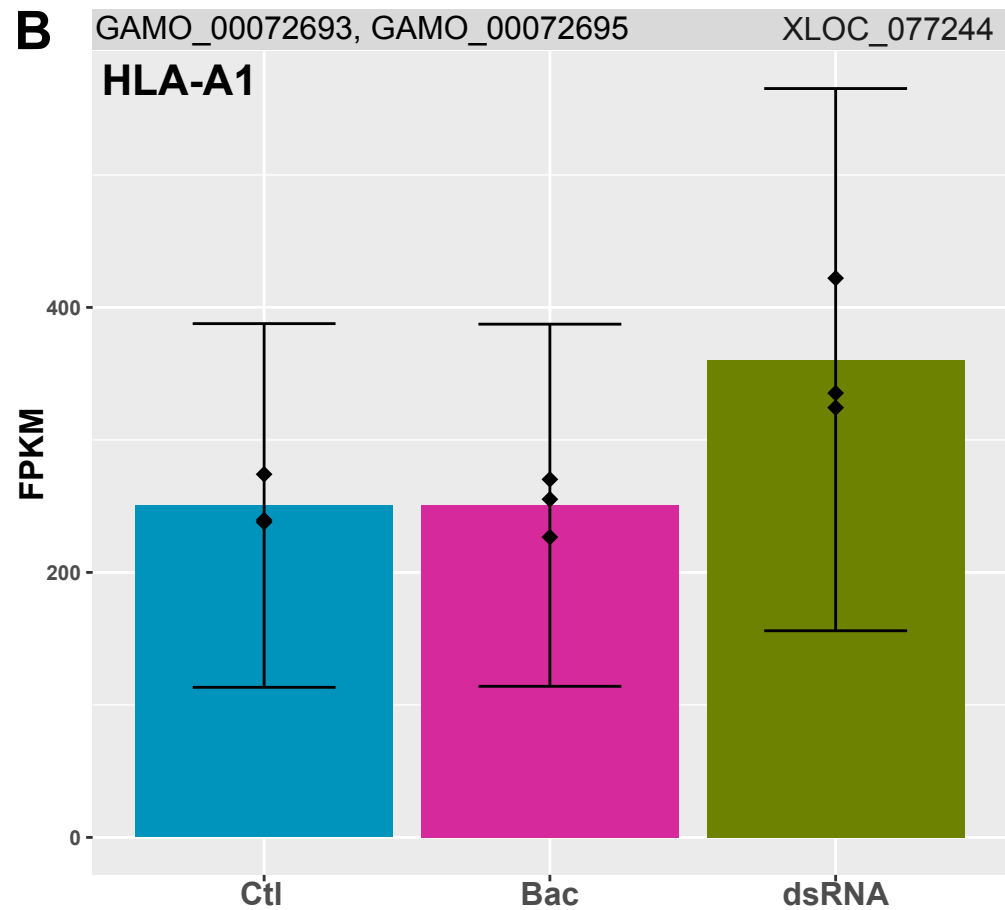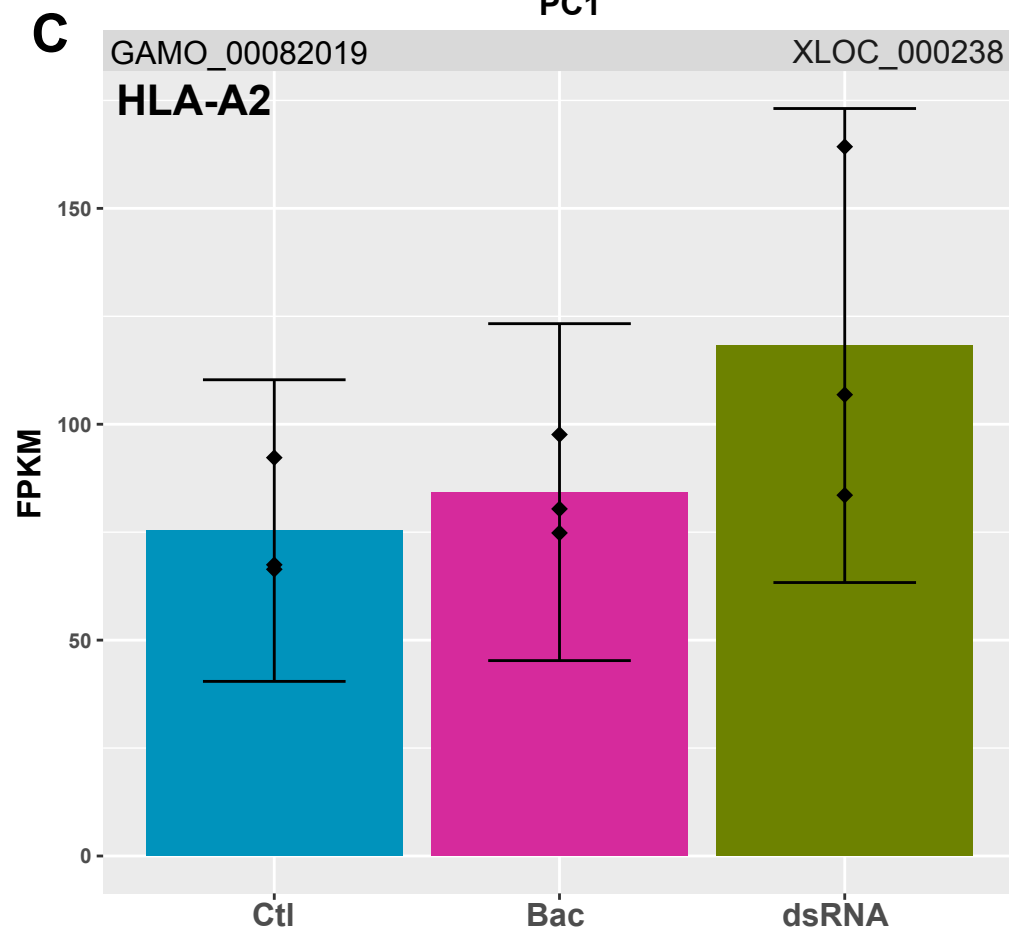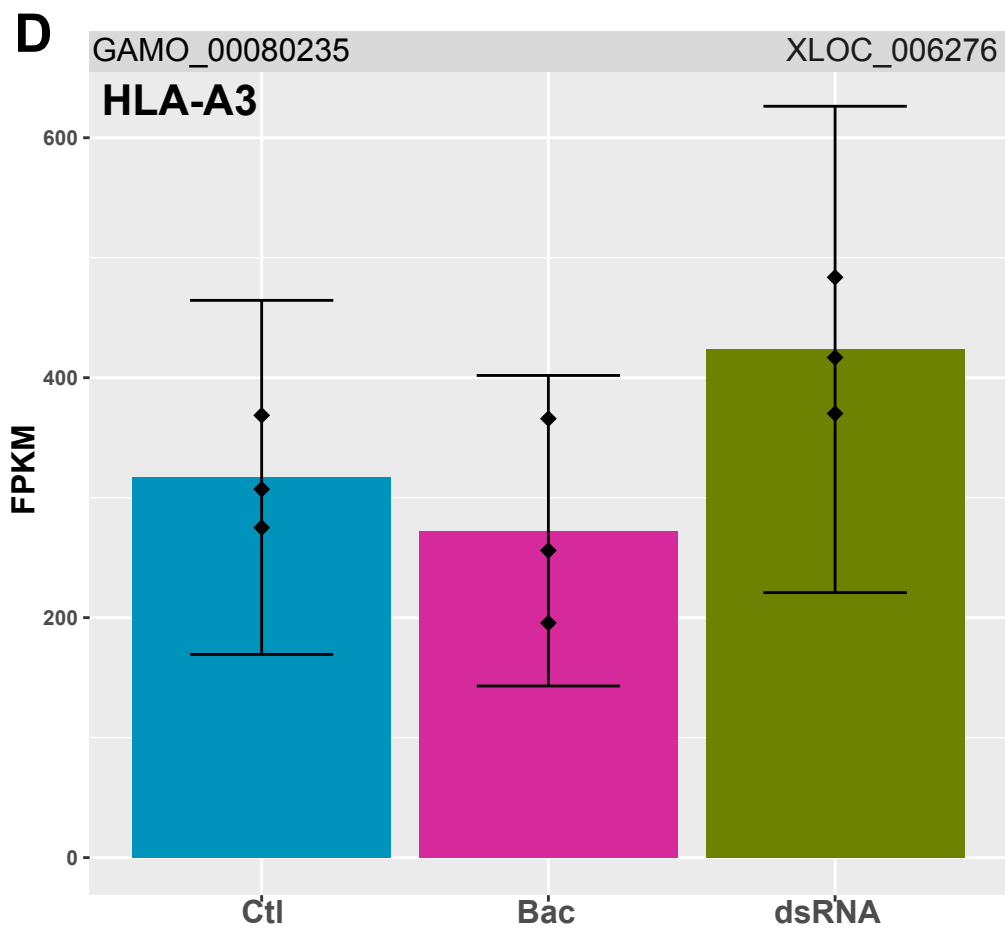

Supplement: Supplementary file 2 [file DataSheet_2.pdf]

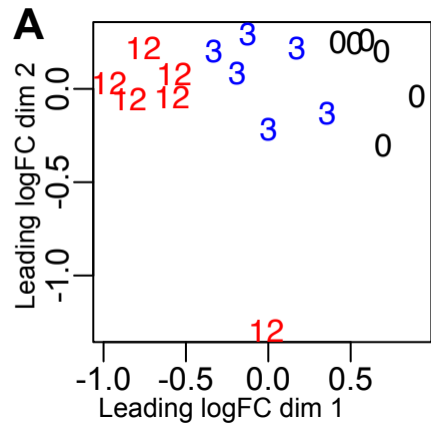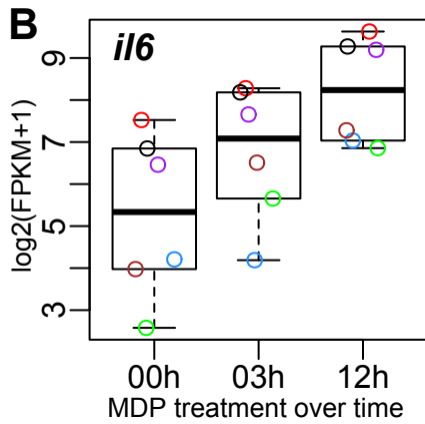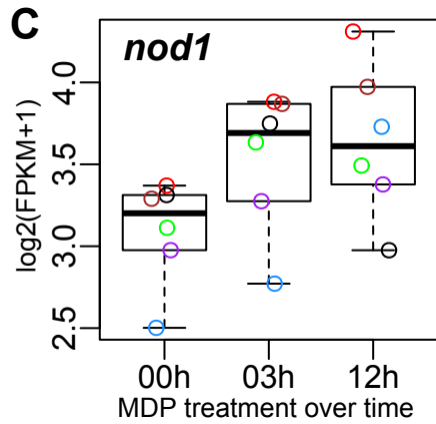

Supplement: Supplementary file 3 [file DataSheet_3.pdf]

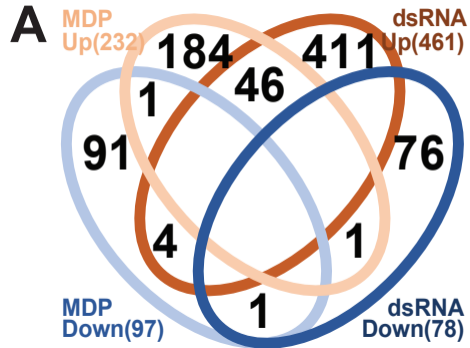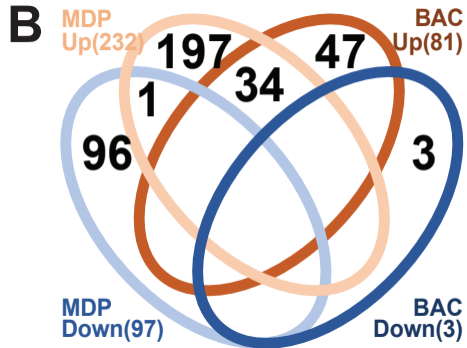

Supplement: Supplementary file 4 [file DataSheet_4.pdf]

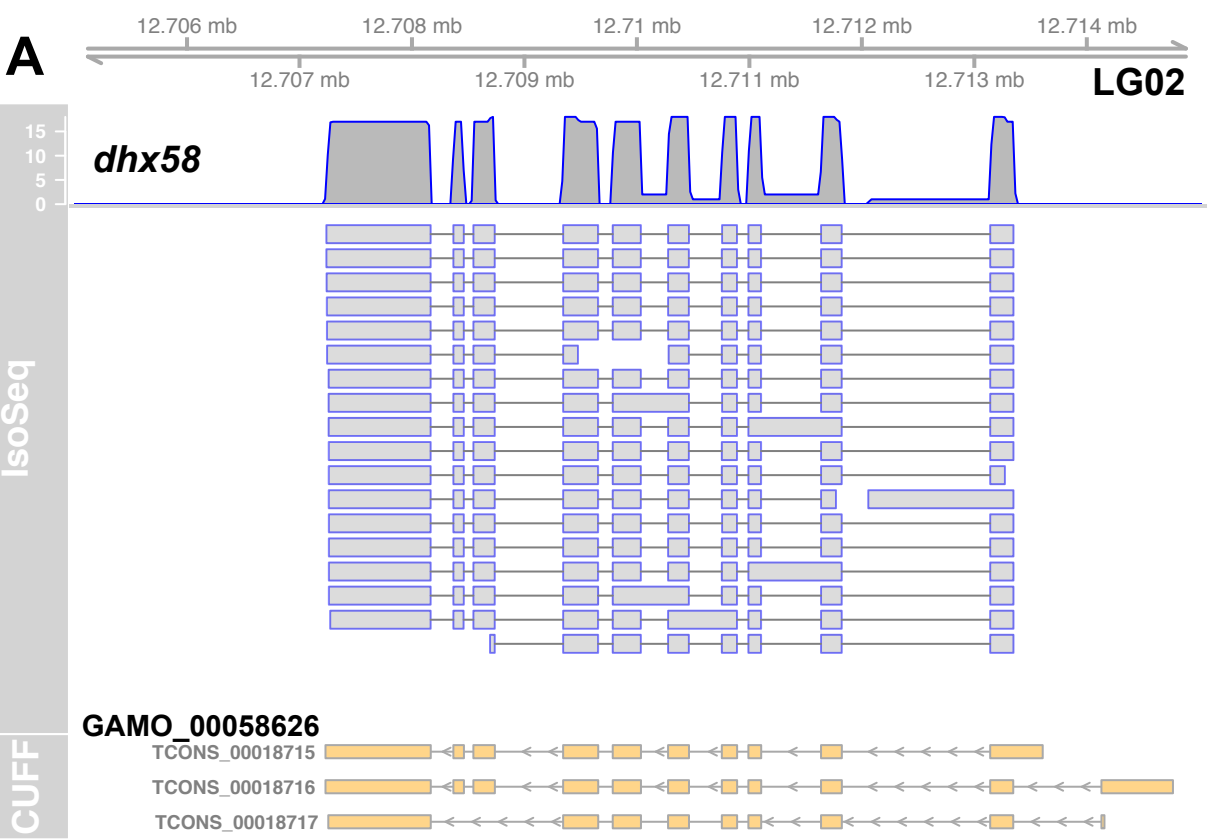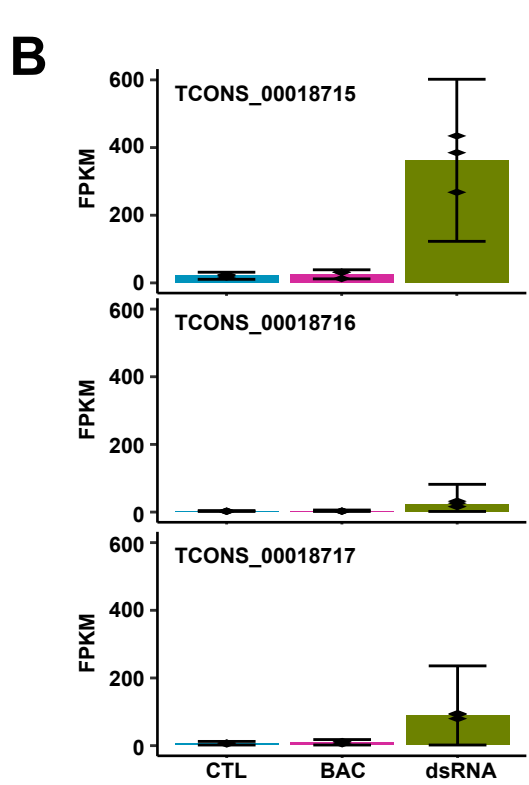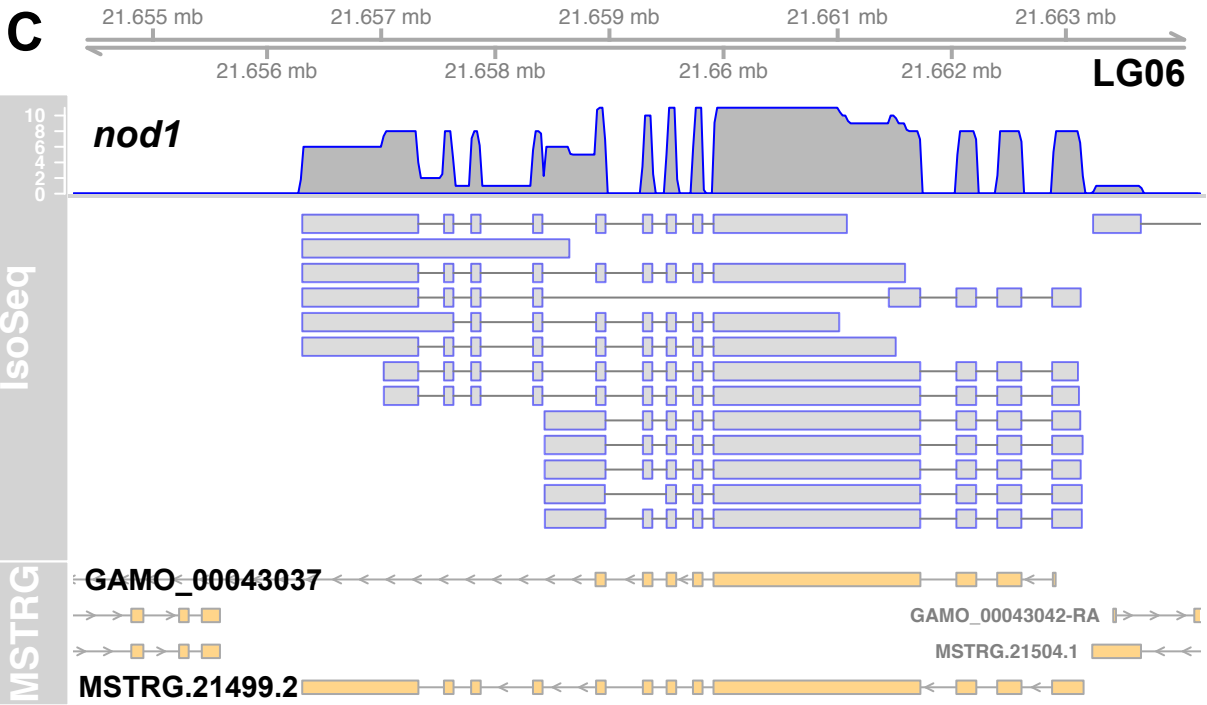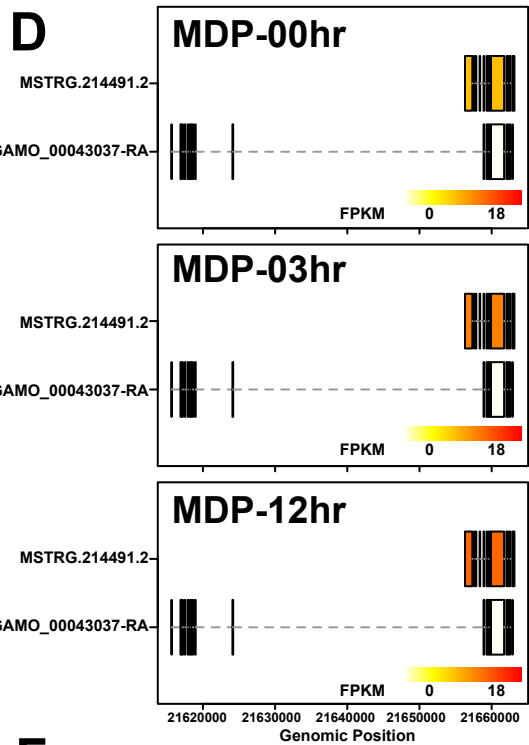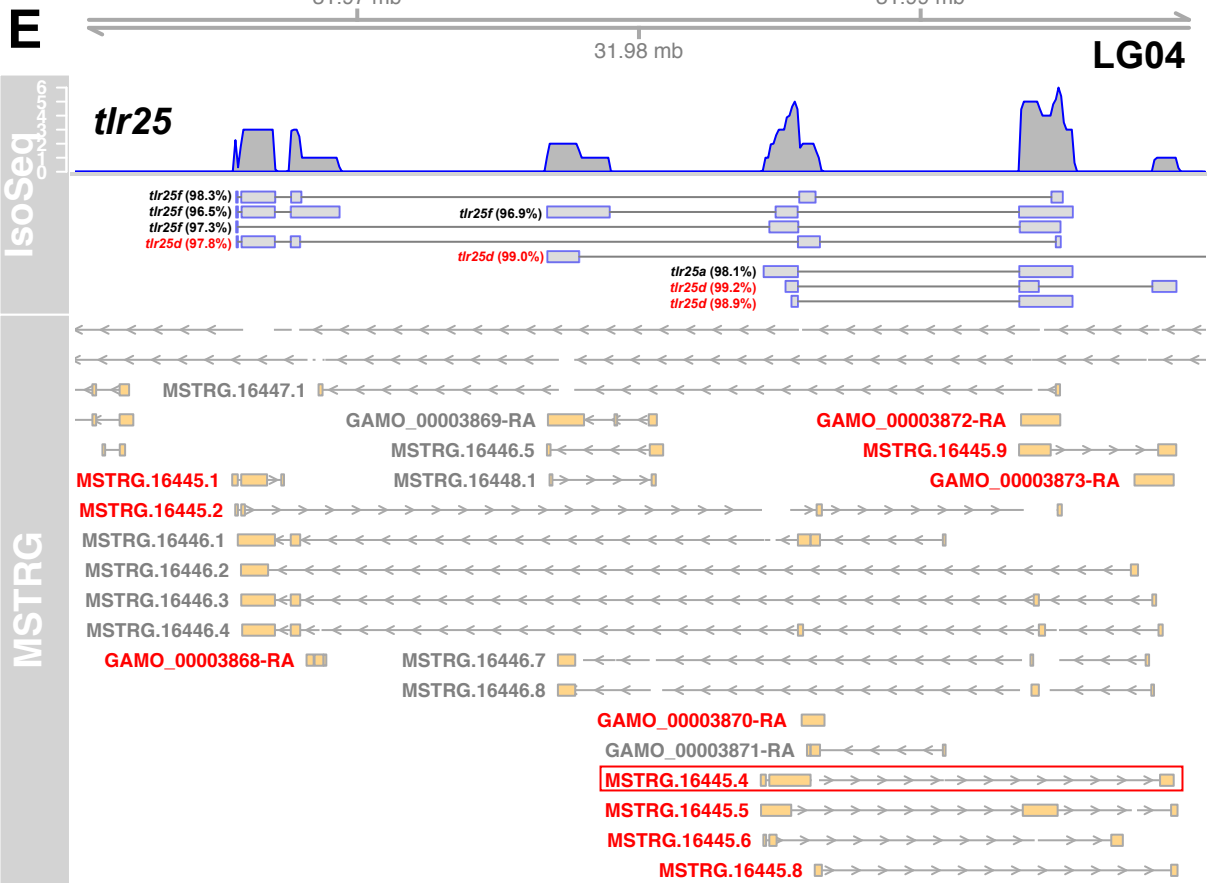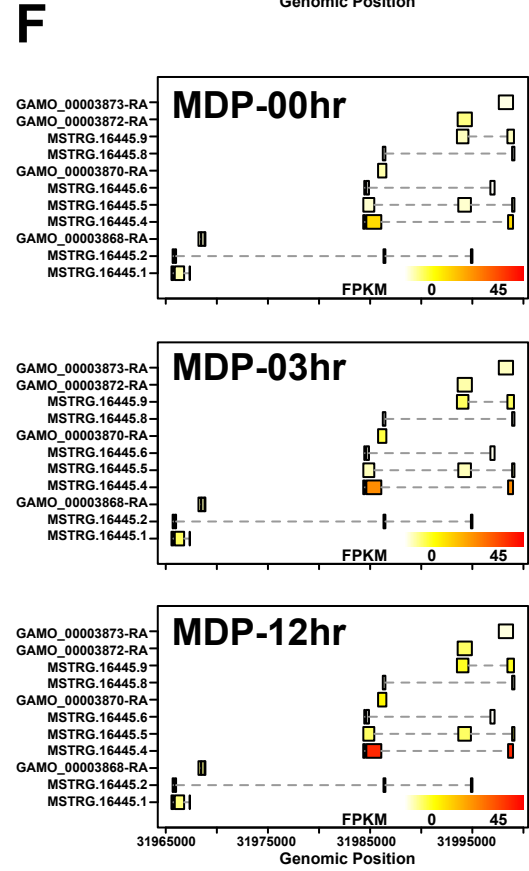

Supplement: Supplementary file 6 [file DataSheet_6.pdf]

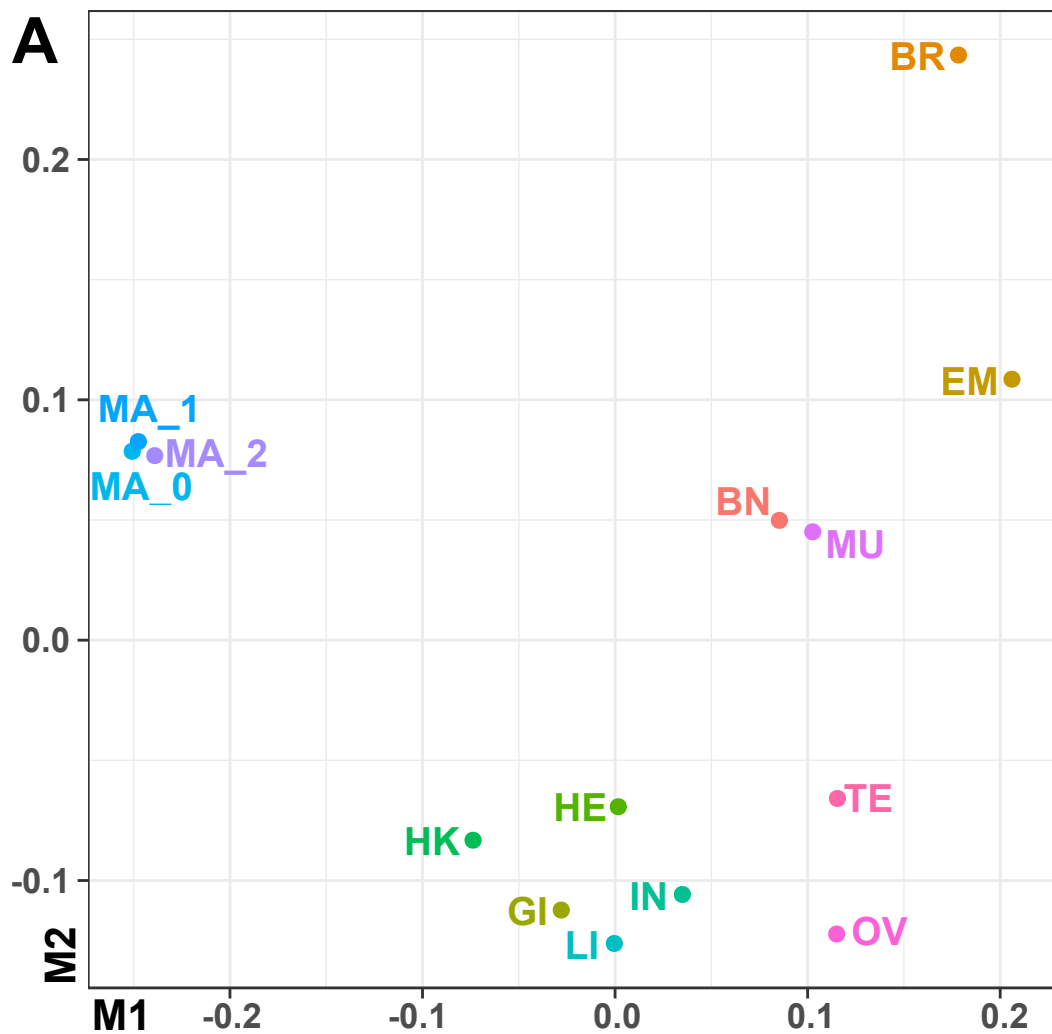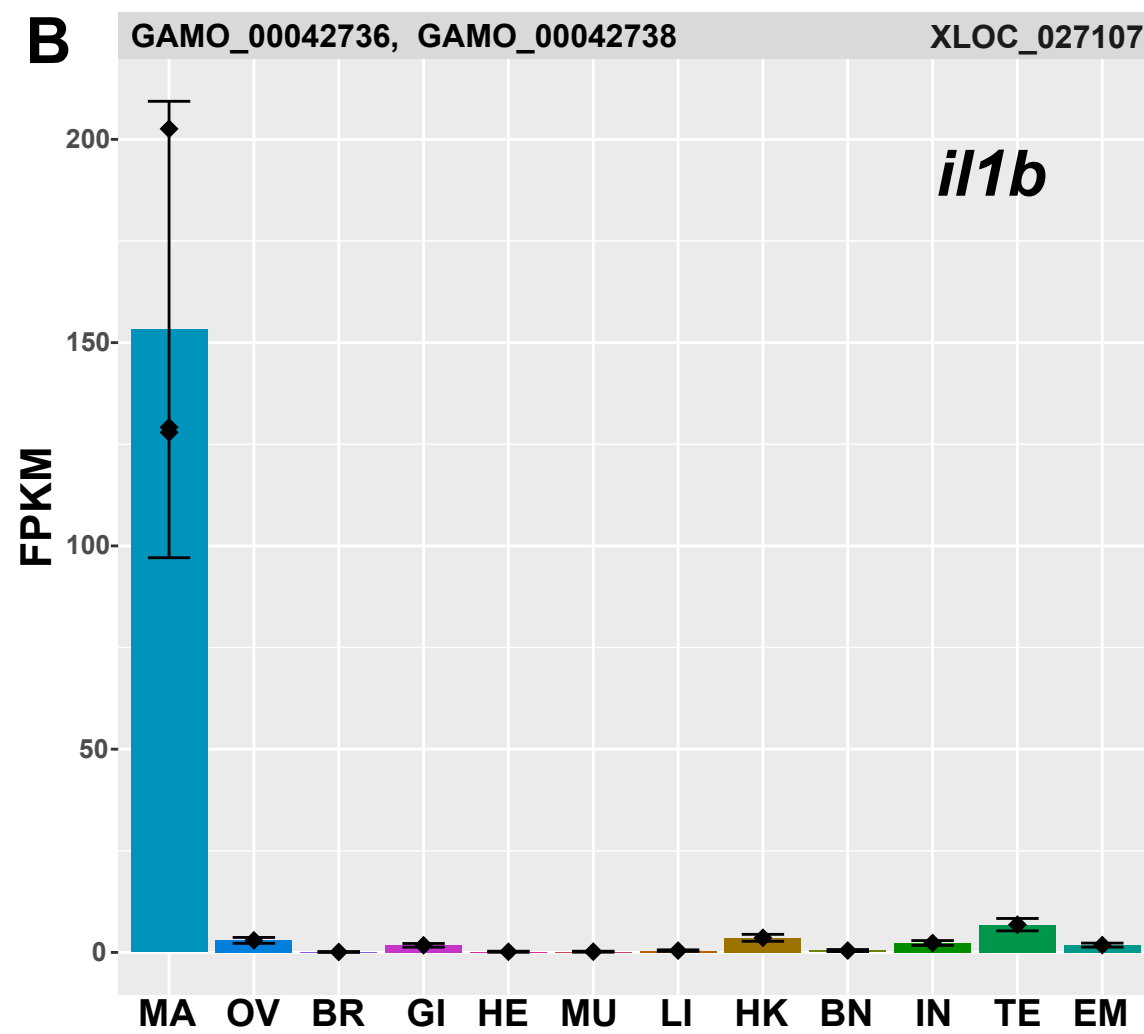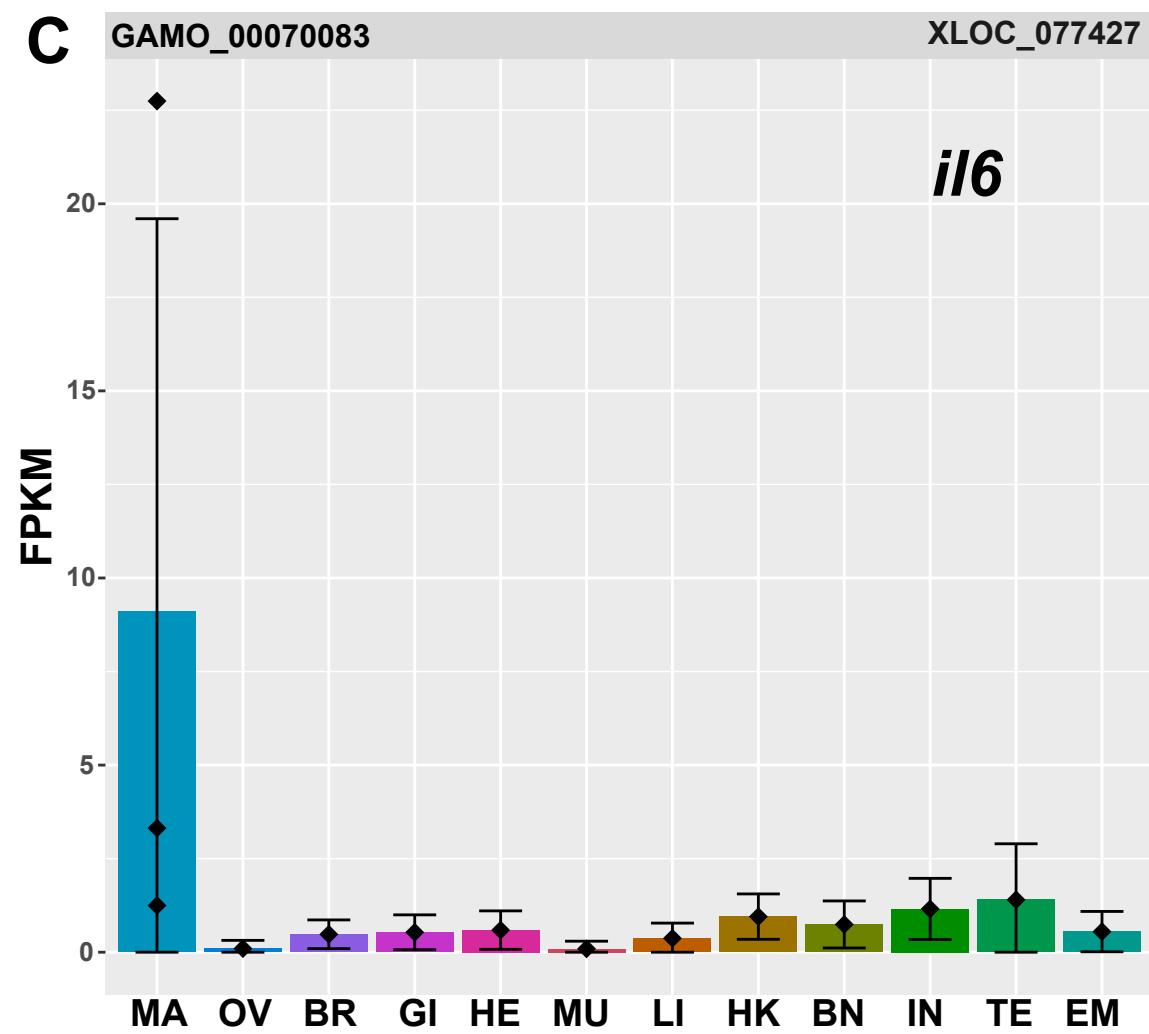

Supplement: Supplementary file 7 [file DataSheet_7.pdf]
