# Supplementary material for: Innovation in Nucleotide-Binding Oligomerization-Like Receptor and Toll-Like Receptor Sensing Drives the Major Histocompatibility Complex-II Free Atlantic Cod Immune System
Source: Front Immunol. 2020 Dec 11;11:609456. doi: 10.3389/fimmu.2020.609456 (PMC7759675; doi:10.3389/fimmu.2020.609456)
Supplement: Supplementary file 5 [file DataSheet_5.pdf]

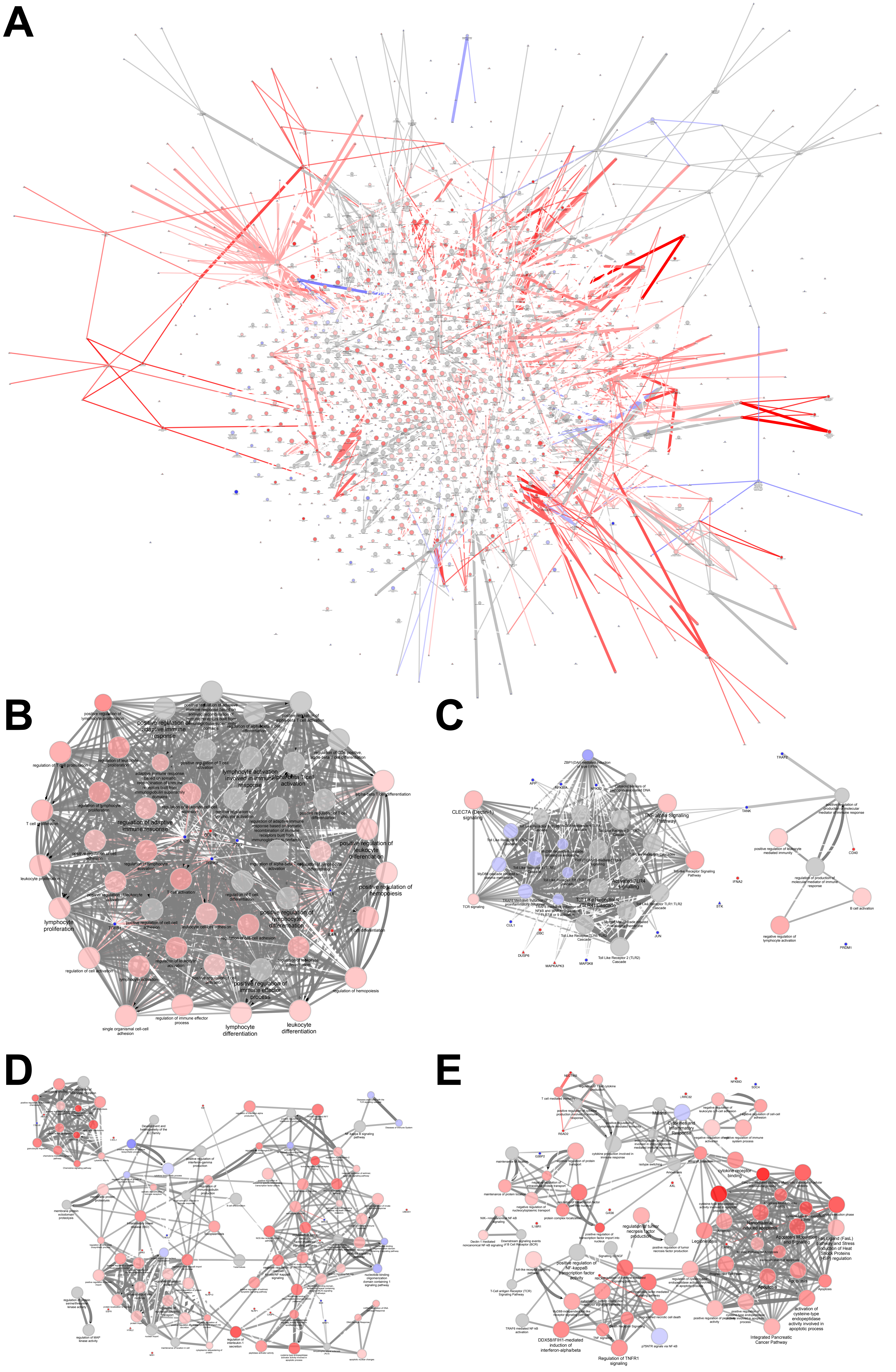
A
B
positive regulation of lymphocyte proliferation
positive regulation of adaptive immune response
positive regulation of adaptive immune response based on somatic recombination of immune receptors built from immunoglobulin superfamily domains
regulation of alpha-beta T cell differentiation
positive regulation of alpha-beta T cell activation
regulation of T cell proliferation
regulation of leukocyte proliferation
positive regulation of T cell activation
regulation of CD4-positive, alpha-beta T cell differentiation
lymphocyte activation involved in immune response
alpha-beta T cell activation
adaptive immune response based on somatic recombination of immune receptors built from immunoglobulin superfamily domains
alpha-beta T cell differentiation
regulation of lymphocyte proliferation
regulation of leukocyte cell-cell adhesion
positive regulation of lymphocyte activation
T cell proliferation
regulation of adaptive immune response
positive regulation of T cell differentiation
regulation of adaptive immune response based on somatic recombination of immune receptors built from immunoglobulin superfamily domains
positive regulation of cell adhesion
IL12B
IFNG
positive regulation of leukocyte differentiation
regulation of lymphocyte activation
regulation of alpha-beta T cell activation
positive regulation of lymphocyte differentiation
leukocyte proliferation
positive regulation of hemopoiesis
positive regulation of leukocyte activation
T cell activation
regulation of T cell differentiation
HLX
lymphocyte proliferation
TGFB1
positive regulation of cell-cell adhesion
leukocyte cell-cell adhesion
positive regulation of lymphocyte differentiation
T cell differentiation
IL4R
regulation of cell-cell adhesion
regulation of cell activation
regulation of leukocyte activation
regulation of leukocyte differentiation
lymphocyte activation
positive regulation of cell activation
positive regulation of immune effector process
regulation of hemopoiesis
single organismal cell-cell adhesion
regulation of immune effector process
lymphocyte differentiation
leukocyte differentiation
C
ZBP1(DAI) mediated induction of type I IFNs
TRAF2
APP
NFKBIA
NFKB2
Cytosolic sensors of pathogen-associated DNA
CLEC7A (Dectin-1) signaling
TNF alpha Signaling Pathway
TANK
positive regulation of production of molecular mediator of immune response
Toll Like Receptor 10 (TLR10) Cascade
Toll Like Receptor 3 (TLR3) Cascade
positive regulation of leukocyte mediated immunity
CD40
Toll-like Receptor Cascades
Toll-like Receptor Signaling Pathway
IFNA2
regulation of production of molecular mediator of immune response
Activated TLR4 signalling
TCR signaling
Toll Like Receptor 4 (TLR4) Cascade
Toll Like Receptor TLR1:TLR2 Cascade
BTK
B cell activation
CUL1
UBC
JUN
Toll Like Receptor TLR6:TLR2 Cascade
negative regulation of lymphocyte activation
DUSP6
MAPKAPK3
MAP3K8
Toll Like Receptor 2 (TLR2) Cascade
PRDM1
D
E
NECTIN2
regulation of T cell cytokine production
T cell mediated immunity
NFKBID
SDC4
LRRC32
Malaria
Cytokines and Inflammatory Response
RSAD2
cytokine production involved in immune response
isotype switching
cytokine receptor binding
Allograft Rejection
Amoebiasis
negative regulation of protein localization
maintenance of protein location
AXL
GAS6
Legionellosis
regulation of tumor necrosis factor production
positive regulation of tumor necrosis factor production
Nanomaterial induced apoptosis
Apoptosis Modulation and Signaling
Fas Ligand (FasL) pathway and Stress induction of Heat Shock Proteins (HSP) regulation
IL18R1
NIK-->noncanonical NF-kB signaling
Dectin-1 mediated noncanonical NF-kB signaling
Downstream signaling events of B Cell Receptor (BCR)
positive regulation of NF-kappaB transcription factor activity
Signaling by NGF
Apoptosis
toll-like receptor signaling pathway
T-Cell antigen Receptor (TCR) Signaling Pathway
activation of cysteine-type endopeptidase activity involved in apoptotic process
TRAF6 mediated NF-kB activation
DDX58/IFIH1-mediated induction of interferon-alpha/beta
TNF signaling
Death Receptor Signaling
p75NTR signals via NF-kB
Regulation of TNFR1 signaling
Integrated Pancreatic Cancer Pathway
